# Density-Potential Functional Theoretic (DPFT) Schemes of Modeling Reactive Solid–Liquid Interfaces

**DOI:** 10.1021/acsphyschemau.5c00071

**Published:** 2025-10-02

**Authors:** Xiwei Wang, Jun Huang

**Affiliations:** † Institute of Energy Technologies, IET-3: Theory and Computation of Energy Materials, 28334 Forschungszentrum Jülich GmbH, 52425 Jülich, Germany; ‡ Theory of Electrocatalytic Interfaces, Faculty of Georesources and Materials Engineering, RWTH Aachen University, 52062 Aachen, Germany

**Keywords:** solid−liquid interfaces, computational electrochemistry, electrical double layer, density-potential functional theory, double-layer capacitance

## Abstract

Simulating electron transfer at reactive solid–liquid interfaces under constant electrochemical potentials of the constituents (electrons, ions, solvent, etc.) is crucial to understanding the formation, function, and failure of electrochemical devices and beyond. Albeit largely accurate in describing the breaking and formation of chemical bonds at solid surfaces, existing methods based on Kohn–Sham density functional theory (DFT) are unsatisfactory in system consistency, namely, simulating the solid–liquid interface under grand-canonical conditions, as well as in scaling up the simulation due to its high computational cost. Herein, to improve the system consistency and computational efficiency, we develop density-potential functional theoretic (DPFT) schemes out of first-principles, drawing upon ideas of Kohn–Sham DFT, orbital-free DFT, frozen density embedding theory, and tight-binding DFT. The proposed DPFT transforms an all-atom, Kohn–Sham DFT description of the nonreactive electrolyte solution into a coarse-grained, field-based description, while retaining a Kohn–Sham DFT description for the reactive subsystem. As a proof of concept, a one-dimensional, orbital-based DPFT model is presented. To reduce the computational cost further, the solid electrode can be described using orbital-free DFT, resulting in orbital-free DPFT models. On the conceptual level, the physical meaning of potential in DPFT is examined. On the application level, the merits and shortcomings of each scheme are compared. This work lays a theoretical basis for DPFT schemes of modeling (reactive) solid–liquid interfaces.

## Graphical abstract

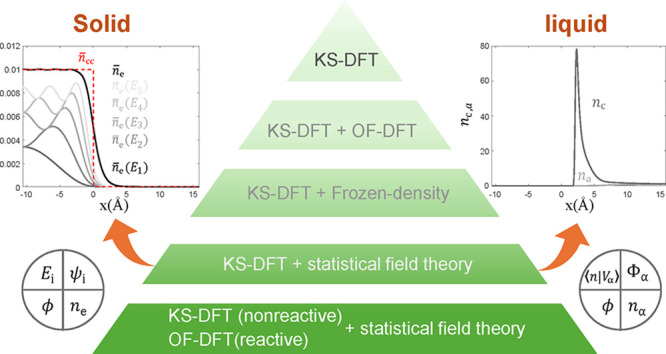

## Introduction

Solid–liquid interfaces are the key functional component in supercapacitors,^1–3^ batteries,^4–6^ fuel cells,^7–9^ (photo)­electrolysis cells,^10–12^ nanofluidic devices^13,14^ etc. Examples include the carbon-aqueous solution interfaces in supercapacitors,^15,16^ lithium-nonaqueous solution interfaces in next-generation batteries,^17,18^ platinum-aqueous solution interfaces in polymer electrolyte fuel cells etc.^19,20^ Along with remarkable progress in experimental characterization of these interfaces, theory and modeling are integral to fundamental understanding.^6,21^ Traditionally, models are used in interpretation of experimental capacitance curves, bringing forth a wealth of microscopic understanding of the structure of solid–liquid interfaces.^22–24^ In so-called nontraditional approaches, theory and modeling are often needed to deconvolute weak signal of solid–liquid interfaces from much stronger noise from the two adjacent bulk phases.^25–27^

Currently, a standard approach to simulate solid–liquid interfaces is nonexistent, according to recent comprehensive reviews on this topic.^28,29^ Therefore, theory and modeling of solid–liquid interfaces is a vibrant research field with a strong focus on method development. Methods in various flavors are being actively developed, including but not limited to density-functional theory (DFT) models without any solvent^30^ or with a continuum description of the electrolyte solution,^31–37^ DFT based molecular dynamics (MD) simulations,^38–40^ classical MD simulations,^3,41–43^ hybrid quantum mechanics/molecular mechanics (QM/MM) simulations,^44–47^ and continuum models.^23,24,48–51^ Each method has its own advantages and disadvantages, as critically reviewed by Schwartz et al.^28^ and Ringe et al.^29^

We and others have been developing density-potential functional theoretic (DPFT) methods to efficient modeling of solid–liquid interfaces.^52–59^ As for the solid phase, the existing DPFT scheme adopts an orbital-free DFT for delocalized electrons, circumventing expensive calculations of Kohn–Sham wave functions.^60–63^ As for the electrolyte solution, DPFT adopts a coarse-grained, field-theoretic treatment, like the continuum/implicit solvation models but with important differences. Specifically, the most recent version of DPFT incorporates short-range correlations between solvent molecules and those between solvent and ions,^64–66^ which are often neglected in continuum/implicit solvation models on the mean-field level.^55^ Consequently, the DPFT model can capture oscillatory distributions of electrostatic potential and ion densities in the liquid phase, while continuum/implicit solvation models give rise to monotonic distributions of these quantities. Moreover, DPFT allows us to simulate solid–liquid interfaces with open boundaries that allow exchange of particles, a generic term for electrons, ions and solvent molecules in the system, with reservoirs of these particles held at constant electrochemical potentials. The grand canonical nature of DPFT ensures system consistency in computer simulations of real-world experiments. Finally, the leap in efficiency and the preservation of system consistency, together, bring opportunities to simulate solid–liquid interfaces at nanoparticles that are beyond reach of DFT and even cheaper classical MD methods, see a recent example by Zhang et al.^67^

The purpose of this paper is 3-fold. First, we consolidate the theoretical basis of DPFT by conducting a systematic derivation starting from a first-principles theory. In so doing, we formally define all approximations from many-electron Schrödinger equation all the way down to DPFT. Second, we examine the origin of the potential in DPFT. Specifically, we address the conceptual question: Why do density and potential have the same status in DPFT, while potential is expressed as an integral function of electron density and has an inferior status in Kohn–Sham DFT? Third, we extend DPFT from the orbital-free scheme in previous works^52–59^ to orbital-based schemes. This extension paves the way for modeling reactive solid–liquid interfaces under grand canonical conditions. A proof-of-concept example of orbital-based DPFT is presented.

The reminder of this paper is organized as follows. We first give a short summary of the various simulation schemes derived from the same starting point with different levels of approximation. Then, we present a detailed derivation of these schemes. Readers who are not interested in those technical details may skip this part. Next, we present an implementation of the orbital-based schema in a 1D model. After that, different schemes are compared in terms of accuracy, system consistency, computational efficiency and transferability. In the end, we will conclude the main results.

## Short Summary

Five schemes of modeling electrified solid–liquid interfaces are derived from nonrelativistic Schrödinger equation of many electrons by applying a hierarchy of approximations. Key features of these schemes are summarized in Figure 1. They differ in the treatment of the reactive subsystem consisting of the solid electrode and adsorbates on the solid surface, and the nonreactive liquid environment, as depicted in Figure 1a. These schemes are compared in four aspects: accuracy, system consistency, computational efficiency and transferability in Figure 1b.

**Figure 1:**
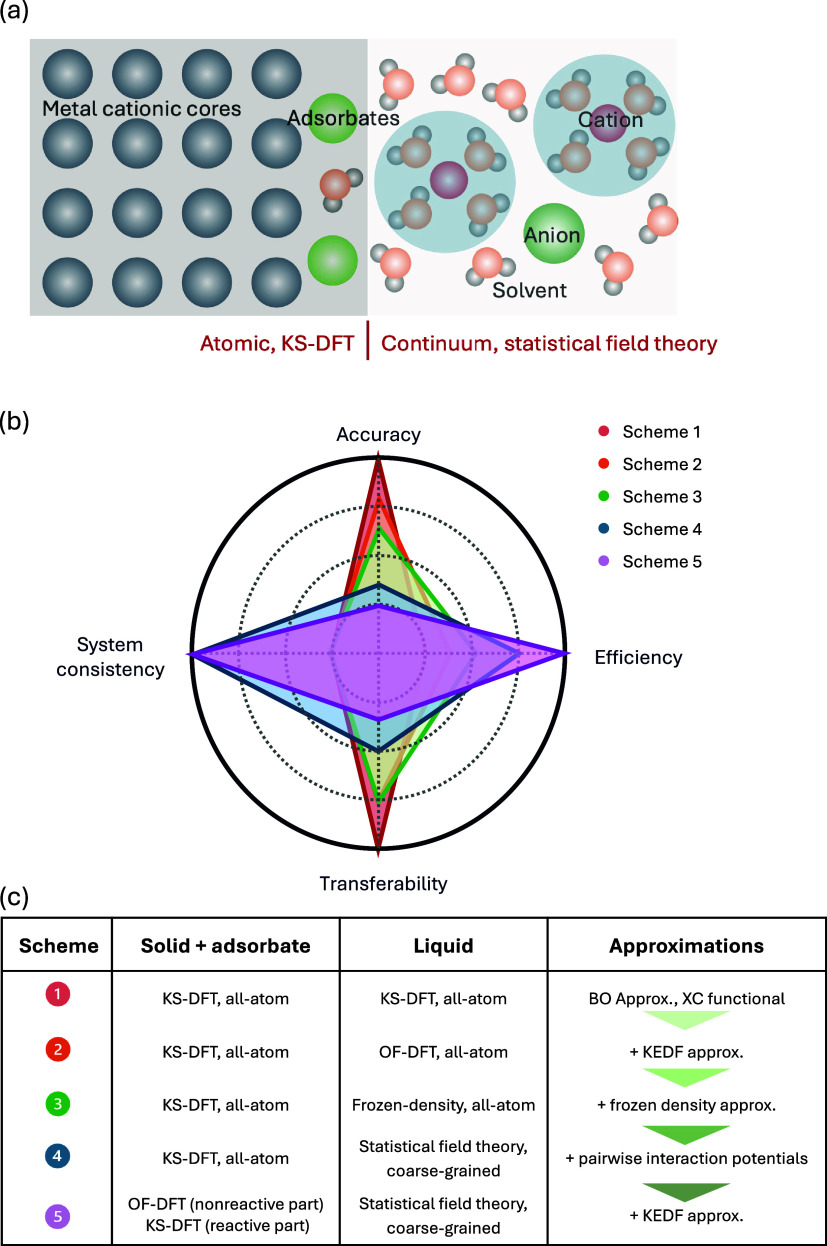
Comparison of five schemes of modeling metal-solution interfaces. (a) Schematic of the metal-solution interfaces with adsorbates on the metal surface. (b) Performances of five approximation schemes which are detailed in (c).

The first scheme applies Kohn–Sham DFT to compute the electronic structure of the whole interface with an all-atom description. Main approximations include the Born–Oppenheimer approximation and approximations in the exchange-correlation (XC) functional.

The second scheme employs orbital-free DFT to describe the nonreactive liquid environment, bringing approximations in the kinetic energy functional. This also brings forth the problem of handling the nonadditivity of kinetic energy functional in the boundary region between KS-DFT and OF-DFT parts.

The third scheme does not solve the electronic density in the nonreactive liquid environment; instead, it uses a frozen density description to calculate the two-body interaction potentials.

The fourth scheme represents a different category of hybrid particle-field theoretic methods, introducing auxiliary potentials to describe the two-body interactions in the nonreactive liquid environment.^68,69^ The transformation from particle-based theoretic methods to hybrid particle-field theoretic methods allows us to properly treat the matter exchange between the system and the environment.

The fifth scheme is different from the fourth scheme, intended to reduce the computational cost by using OF-DFT to simulate most atoms in the solid phase that are not directly involved in the surface reaction.

## Systematic Derivation

### Kohn–Sham Scheme

We consider a system of *N*_e_ electrons and *N*_n_ nuclei of multiple types that are not distinguished for the moment. Following the convention of Gross et al.,^70,71^ we denote the coordinates of electrons {**r**_i_}, the mass of electrons *m*_e_, the coordinates of nuclei {**R**_i_}, and the masses of nuclei {*M*_*i*_}. We use the SI units system throughout the paper unless otherwise noted. Operators acting on wave functions are denoted with hat notations.

The nonrelativistic Hamiltonian of the system is,

$$\hat{H}={\hat{T}}_{e}+{\hat{T}}_{n}+{\hat{V}}_{\mathrm{nn}}+{\hat{V}}_{\mathrm{ne}}+{\hat{V}}_{\mathrm{ee}}+{\hat{U}}_{n}+{\hat{U}}_{e}$$ 1

where the kinetic energy terms are,

$${\hat{T}}_{n}=-\sum_{i=1}^{N_{n}}\frac{\hbar^{2}}{2M_{i}}\nabla_{R_{i}}^{2}$$ 2

$${\hat{T}}_{e}=-\sum_{i=1}^{N_{e}}\frac{\hbar^{2}}{2m_{e}}\nabla_{r_{i}}^{2}$$ 3

for nuclei and electrons, respectively.

The nucleus–nucleus, electron–electron, nucleus-electron Coulomb interaction terms are,

$${\hat{V}}_{\mathrm{nn}}=\frac{1}{4\pi \epsilon_{0}}\sum_{i}\sum_{j<i}\frac{Z_{i}Z_{j}e^{2}}{\left|R_{i}-R_{j}\right|}$$ 4

$${\hat{V}}_{\mathrm{ee}}=\frac{1}{4\pi \epsilon_{0}}\sum_{i}\sum_{j<i}\frac{e^{2}}{\left|r_{i}-r_{j}\right|}$$ 5

$${\hat{V}}_{\mathrm{ne}}=-\frac{1}{4\pi \epsilon_{0}}\sum_{i}\sum_{j}\frac{Z_{j}e^{2}}{\left|r_{i}-R_{j}\right|}$$ 6

with ϵ_0_ being the vacuum permittivity.

The truly external potential terms, such as the voltage applied to the system, are,

$${\hat{U}}_{n}=\sum_{i=1}^{N_{n}}U_{n}(R_{i})$$ 7

$${\hat{U}}_{e}=\sum_{i=1}^{N_{e}}u_{n}(r_{i})$$ 8

Herein, the external potential operator acts as a multiplicative function in that it does not differentiate or modify the wave function, but just scales it. The hat might be dropped out for these multiplicative operators without incurring any confusion.

No Born–Oppenheimer approximation has been assumed in eq 1; instead, all electronic and nuclear degrees of freedom are described quantum mechanically. The Born–Oppenheimer approximation allows us to separate the wave functions of nuclei and electrons for nuclei are much heavier than the electrons. Therefore, the electronic Hamiltonian becomes,

$${\hat{H}}_{e}={\hat{T}}_{e}+{\hat{V}}_{\mathrm{ne}}+{\hat{V}}_{\mathrm{ee}}+{\hat{U}}_{e}$$ 9

and the energy of the electronic part for a wave function |Ψ⟩ is given by,

$$E_{e}=\langle \Psi |{\hat{H}}_{e}|\Psi \rangle$$ 10

The total energy of the system is given by,

$$E_{\mathrm{tot}}=E_{e}+\sum_{i=1}^{N_{n}}\frac{(p_{i}{)}^{2}}{2M_{i}}+\frac{1}{4\pi \epsilon_{0}}\sum_{i}\sum_{j<i}\frac{Z_{i}Z_{j}e^{2}}{\left|R_{i}-R_{j}\right|}+\sum_{i=1}^{N_{n}}U_{n}(R_{i})$$ 11

with |Ψ⟩ is the electronic wave function as a function of the nuclei coordinates {**R**_i_}, the second term is the kinetic energy of nuclei which are further assumed to be classical with momentums {**p**_i_}, the third term is the repulsive Coulomb interaction energy between nuclei, and the last term is the external potential energy of the nuclei.

In the density functional formalism given by Hohenberg and Kohn,^72^ the electron–nucleus interaction is included, together with the truly external potential *Û*_e_, in an external potential term *V̂*_ext_,

$${\hat{V}}_{\mathrm{ext}}={\hat{V}}_{\mathrm{ne}}+{\hat{U}}_{e}$$ 12

The electronic Hamiltonian ${\hat{H}}_{e}$ is written as,

$${\hat{H}}_{e}={\hat{T}}_{e}+{\hat{V}}_{\mathrm{ee}}+{\hat{V}}_{\mathrm{ext}}$$ 13

Hohenberg and Kohn^72^ and later Levy^73^ proved that there is a one-to-one relationship between *V̂*_ext_ and the ground-state |Ψ⟩, and that there is a one-to-one relationship between |Ψ⟩ and the ground state electron density *n*. Therefore, the ground state energy of *Ĥ*_e_ is a functional of the electron density,

$$E_{e,\mathrm{GS}}=\int drV_{\mathrm{ext}}(r)n(r)+F[n]$$ 14

where *F*[*n*] is the universal functional that is general to any *V̂*_ext_, formally defined as,

$$F[n]=\langle \Psi_{n}|{\hat{T}}_{e}+{\hat{V}}_{\mathrm{ee}}|\Psi_{n}\rangle$$ 15

where |Ψ*_n_* ⟩ is the ground state wave function corresponding to the ground state density *n*.

Kohn and Sham proposed a decomposition scheme of calculating *E*_e, GS_ involving a fictitious electronic system with the same density *n*, however, in the absence of electron–electron interactions other than the classical coulomb interactions.^74^ The Kohn–Sham scheme transforms *F*[*n*] to

$$F[n]=T_{s}[n]+\frac{e^{2}}{8\pi \epsilon_{0}}\int drdr^{\prime }\frac{n(r)n(r^{\prime })}{\left|r-r^{\prime }\right|}+E_{\mathrm{xc}}[n]$$ 16

where *T*_s_[*n*] is the kinetic energy of noninteracting electrons in the fictitious scheme, the second term is the classical Coulomb energy between electrons, and the last term is the exchange-correlation term. *E*_xc_[*n*] accounts for both the difference between true kinetic energy and *T*_s_[*n*], and the difference between true Coulomb energy ⟨Ψ*_n_* | *V̂*_ee_ | Ψ*_n_* ⟩ and the classical Coulomb energy.

*T*_s_[*n*] is given by,

$$T_{s}[n]=\sum_{i}f_{i}\langle \psi_{i}|-\frac{\hbar^{2}}{2m_{e}}\nabla_{r}^{2}|\psi_{i}\rangle$$ 17

where |ψ_*i*_⟩ is the *i*th orbital of the noninteracting system, and *f*_*i*_ is the fractional occupancy. |ψ_*i*_⟩ is found from solving the Schrödinger equation of the noninteracting system,

$$\left(-\frac{\hbar^{2}}{2m_{e}}\nabla_{r}^{2}+V_{\mathrm{eff}}(r)\right)|\psi_{i}\rangle =\epsilon_{i}|\psi_{i}\rangle$$ 18

with the effective potential given by,

$$V_{\mathrm{eff}}(r)=\frac{e^{2}}{4\pi \epsilon_{0}}\int dr^{\prime }\frac{n(r^{\prime })}{\left|r-r^{\prime }\right|}+V_{\mathrm{ext}}(r)+\frac{\delta E_{\mathrm{xc}}[n]}{\delta n}$$ 19

where the last term is a functional derivative of the exchange-correlation term.

The spin variable is neglected in the current formalism. Spin-dependent Kohn–Sham scheme is well documented in the literature, e.g., the book by Dreizler and Gross.^75^

The occupancy *f*_*i*_ is given by,

$$f_{i}=\frac{1}{1+\exp\{\beta (\epsilon_{i}-{\mathrm{\mu ̃}}_{e})\}}$$ 20

where β^–1^ = *k*_B_ *T* is the inverse temperature, μ̃_e_ is the electrochemical potential of electrons.

The entropy of noninteracting electrons is given by, for instance, in ref 36.

$$S_{e}[n]=-k_{B}\sum_{i}\{f_{i}\ln\;f_{i}+(1-f_{i})\ln(1-f_{i})\}$$ 21

The electron density is thus given by,

$$n(r)=\sum_{i}f_{i}|\psi_{i}(r){|}^{2}$$ 22

Substituting eq 14 and 16 into eq 11, the total ground state energy of the canonical ensemble is, within the Kohn–Sham scheme, expressed as,

$$E_{\mathrm{tot},\mathrm{GS}}=\{T_{s}[n]+E_{\mathrm{xc}}[n]\}+\sum_{i=1}^{N_{n}}\frac{(p_{i}{)}^{2}}{2M_{i}}+\frac{e^{2}}{8\pi \epsilon_{0}}\int drdr^{\prime }\frac{n(r)n(r^{\prime })}{\left|r-r^{\prime }\right|}+\frac{1}{4\pi \epsilon_{0}}\sum_{i}\sum_{j<i}\frac{Z_{i}Z_{j}e^{2}}{\left|R_{i}-R_{j}\right|}+\int drV_{\mathrm{ext}}(r)n(r)+\sum_{i=1}^{N_{n}}U_{n}(R_{i})$$ 23

The first two terms in the curly brackets are universal functionals describing quantum mechanical interactions between electrons. The third term represents the kinetic energy of classical nuclei. The remaining terms describe classical Coulomb interactions and external potential effects. Note that the classical Coulomb interaction between electrons and nuclei is included in *V*_ext_(**r**), in addition to the truly external potential applied to electrons.

As well-known, the cost of computing the Kohn–Sham scheme in the standard manner grows cubically with the number of orbitals, or approximately, *N*_e_. The high computational cost hinders its application to systems beyond hundreds of atoms. Moreover, it is inefficient to statistically sample the configuration of the nuclei in the liquid phase. Reducing the computational cost motivates us to have a closer look at the studied system and apply, when appropriate, additional approximations that will not severely impair accuracy.

### Hybrid Kohn–Sham and Orbital-Free Scheme

Most electrochemical reactions occur in the inner layer of the solid–liquid interface, as shown in Figure 1. Hence, only a small part of the whole system with formation and cleavage of chemical bonds requires an orbital-based description. Instead, the remaining large part could be described on the orbital-free quantum mechanical level or even on the classical level.

Following Wesolowski and Warshel^76^ and many others,^77–79^ we divide the total system into an orbital-based reactive subsystem with an electron density distribution *n*_ob_(**r**) and an orbital-free environment with an electron density distribution *n*_of_(**r**). The division scheme is a key technical point in practical applications, which is not discussed in this paper focusing on conceptual aspects.

The total electron density is the sum of *n*_ob_(**r**) and *n*_of_(**r**),

$$n_{\mathrm{tot}}(r)=n_{\mathrm{ob}}(r)+n_{\mathrm{of}}(r)$$ 24

The total kinetic energy of electrons could be, without losing any generality, expressed as,^76^

$$T_{s}[n_{\mathrm{tot}}]=T_{s}[n_{\mathrm{ob}}(r)]+T_{s,\mathrm{of}}[n_{\mathrm{of}}(r)]+T_{s}^{\mathrm{nadd}}[n_{\mathrm{ob}}(r),n_{\mathrm{of}}(r)]$$ 25

where *T*_s_^nadd^[*n*_ob_(**r**), *n*_of_(**r**)] is the nonadditive term. We note that *T*_s,of_[*n*_of_(**r**)] is an explicit functional of electron density in the orbital-free subsystem, while *T*_s_[*n*_ob_(**r**)] is an implicit functional of electron density and needs to be calculated using the Kohn–Sham scheme, now with a modified effective potential to be determined shortly.

Practical calculations need an approximate *T*_s_^nadd^ that is an explicit functional of *n*_ob_ and *n*_of_. Usually, *T*_s_^nadd^ is obtained from the same functional for *T*_s, of_[*n*_of_(**r**)],^76,77^

$$T_{s}^{\mathrm{nadd}}[n_{\mathrm{ob}},n_{\mathrm{of}}]\approx T_{s,\mathrm{of}}^{\mathrm{nadd}}[n_{\mathrm{ob}},n_{\mathrm{of}}]=T_{s,\mathrm{of}}[n_{\mathrm{tot}}]-T_{s,\mathrm{of}}[n_{\mathrm{ob}}]-T_{s,\mathrm{of}}[n_{\mathrm{of}}]$$ 26

|ψ_*i*_⟩_ob_ of the orbital-based subsystem is found from solving the Schrödinger equation of the noninteracting subsystem,^76,80^

$$\left(-\frac{\hbar^{2}}{2m_{e}}\nabla_{r}^{2}+V_{\mathrm{eff}}^{\mathrm{ob}}(r)\right)|\psi_{i}\rangle_{\mathrm{ob}}=\epsilon_{i}|\psi_{i}\rangle_{\mathrm{ob}}$$ 27

with the effective potential applied on the orbital-based subsystem given by,

$$V_{\mathrm{eff}}^{\mathrm{ob}}(r)=\frac{e^{2}}{4\pi \epsilon_{0}}\int dr^{\prime }\frac{n_{\mathrm{tot}}(r^{\prime })}{\left|r-r^{\prime }\right|}+V_{\mathrm{ext}}+\frac{\delta E_{\mathrm{xc}}[n_{\mathrm{tot}}]}{\delta n_{\mathrm{ob}}}+\frac{\delta T_{s,\mathrm{of}}^{\mathrm{nadd}}[n_{\mathrm{ob}},n_{\mathrm{of}}]}{\delta n_{\mathrm{ob}}}$$ 28

where the approximate, explicit *T*_s, of_^nadd^ defined in eq 26 is used here.

Now the total grand state energy is given by

$$\begin{array}{ll} E_{\mathrm{tot},\mathrm{GS}} & =T_{s}[n_{\mathrm{ob}}]+T_{s,\mathrm{of}}[n_{\mathrm{of}}]+T_{s,\mathrm{of}}^{\mathrm{nadd}}[n_{\mathrm{ob}},n_{\mathrm{of}}]+E_{\mathrm{xc}}[n_{\mathrm{tot}}]\\ & +\sum_{i=1}^{N_{n}}\frac{(p_{i}{)}^{2}}{2M_{i}}+\frac{e^{2}}{8\pi \epsilon_{0}}\int drdr^{\prime }\frac{n(r)n(r^{\prime })}{\left|r-r^{\prime }\right|}\\ & +\frac{1}{4\pi \epsilon_{0}}\sum_{i}\sum_{j<i}\frac{Z_{i}Z_{j}e^{2}}{\left|R_{i}-R_{j}\right|}+\int drV_{\mathrm{ext}}(r)n(r)+\sum_{i=1}^{N_{n}}U_{n}(R_{i}) \end{array}$$ 29

The electron density in the orbital-free subsystem is found from using the constraint of the electrochemical potential of electrons μ̃_e_,

$${\overset{\sim }{\mu }}_{e}[N_{e},N_{n}]=\frac{\delta E_{\mathrm{tot},\mathrm{GS}}}{\delta n_{\mathrm{of}}}=\frac{\delta T_{s,\mathrm{of}}[n_{\mathrm{of}}]}{\delta n_{\mathrm{of}}}+\frac{\delta T_{s,\mathrm{of}}^{\mathrm{nadd}}[n_{\mathrm{ob}},n_{\mathrm{of}}]}{\delta n_{\mathrm{of}}}+\frac{\delta E_{\mathrm{xc}}[n_{\mathrm{tot}}]}{\delta n_{\mathrm{of}}}+\frac{e^{2}}{4\pi \epsilon_{0}}\int dr^{\prime }\frac{n(r^{\prime })}{\left|r-r^{\prime }\right|}+V_{\mathrm{ext}}$$ 30

There is a key difference between the present hybrid Kohn–Sham and orbital-free theory and the frozen electron density functional theory developed by Wesolowski and Warshel.^76^ In the present scheme, the electron density in the orbital-free region is not frozen but calculated from eq 30. Since orbital-free DFT is a linear scaling method, the computational cost is much lower than the original Kohn–Sham scheme.

If the whole system is described on the orbital-free level, which is a reasonable approximation for cases without chemical bond formation and cleavage at the interface, the electron density is then found from,

$${\tilde{\mu }}_{e}[N_{e},N_{n}]=\frac{\delta T_{s,\mathrm{of}}[n]}{\delta n}+\frac{\delta E_{\mathrm{xc}}[n]}{\delta n}+\frac{e^{2}}{4\pi \epsilon_{0}}\int dr^{\prime }\frac{n(r^{\prime })}{\left|r-r^{\prime }\right|}+V_{\mathrm{ext}}$$ 31

which is a partial differential equation if *T*_s,of_[*n*] and/or *E*_xc_[*n*] contain gradient terms. Thomas–Fermi–von Weizsäcker functional was used for *T*_s,of_ and Perdew–Burke–Ernzerhof (PBE) functional for *E*_xc_ in our previous DPFT models, leading to a second-order partial differential equation of *n*, see eq 50 in ref 55.

### Hybrid Kohn–Sham and Frozen Density Embedding Scheme

The next approximation scheme involves equaling *n*_of_ with the frozen density associated with atoms in the orbital-free subsystem. This removes the need for calculating *n*_of_, facilitating efficient statistical sampling of the electrolyte solution. All electronic polarization in the orbital-free environment is described using empirical parameters, such as the electronic permittivity, in the frozen electron density approximation.

The composition of a nucleus and its associated frozen electron density is termed as pseudoatoms so-called because they might not be electroneutral. A molecule is an assembly of two or more pseudoatoms. Assigning each pseudoatom at **R** *_i_* with a frozen electron density *n*_*i*_^frozen^(**r**, **R**_*i*_), *n*_f_ in the orbital-free environment is then calculated as the sum of all pseudoatoms,

$$n_{f}(r)=\sum_{i=1}^{N_{n}}n_{i}^{\mathrm{frozen}}(r,R_{i})$$ 32

Barker and Sprik used Gaussian functions for *n*_*i*_^frozen^(**r**, **R**_*i*_).^81^

Substituting *n*_f_(**r**) in the orbital-free kinetic energy functional and exchange-correlation functional, we could then calculate the total energy of the orbital-free environment. This is the basic idea of the hybrid Kohn–Sham/frozen density orbital-free method developed by Hodak, Lu, and Bernholc for large biological systems.^78^

### Two-Body Pairwise Interactions in Both Short and Long-Range

Following the idea of semiempirical tight-binding methods,^82–84^ we could transform the total energy of the orbital-free environment into one-body and two-body pairwise terms. The transformation could be made formally exact, as in the perturbation analysis given by Foulkes and Haydock.^82^ Nevertheless, approximation and parametrization are often required in practical applications.

Classical Coulomb interactions between charged particles, a generic term for electrons, nuclei and pseudoatoms, are pairwise and have been separately considered. We only need to approximate the quantum mechanical interactions between pseudoatoms as pairwise interaction potentials, which is more short-ranged compared to the classical Coulomb interactions.

From now on, we will need a more detailed notion of the constituent particles of the system. The orbital-based reaction subsystem is composed of electrons with number density *n*(**r**) and nuclei with number density of *n*_α_(**r**) of which the corresponding operator is given by,

$${\hat{n}}_{\alpha }(r)=\sum_{i=1}^{N_{\alpha }}\delta (r-R_{\alpha ,i})$$ 33

where *N*_α_ is the total number of nuclei of type α in the orbital-based reactive subsystem, and **R**_α, *i*_ is the coordinate of the *i*th nuclei of this type. There are usually multiple types of nuclei in the orbital-based reactive subsystem. Take chemisorption of an ion on a metal surface as an example. There are at least two types of nuclei, namely, the nuclei of the metal atoms and nuclei of the chemisorbed ion. We designate the nuclei of metal atoms collectively as α = M, and the nuclei of adsorbates as α = A.

In a coarse-grained picture, the orbital-free environment is composed of cations and anions with number densities of *n*_c_(**r**) and *n*_a_(**r**), respectively, and solvent molecules with number density of *n*_s_(**r**). We consider only one type of cations and anions and solvent in this work to avoid the notations becoming exceedingly complicated. However, the extension to multiple types of these particles seems straightforward.

The local charge density containing electrons and nuclei in the orbital-based reactive subsystem is collectively expressed as,

$$\rho (r)=-en(r)+\sum_{\alpha =M,A,c,a}Z_{\alpha }en_{\alpha }(r)+(p_{s}\cdot \nabla )n_{s}(r)$$ 34

where *p*_s_ is the dipole moment of solvent molecules, i.e., the vector pointing from the centroid of the positive charge to that of the negative charge within a solvent molecule. The third term (*p*_s_ · ∇)*n*_s_(**r**) in this equation represents the polarization charge density contributed by inhomogeneous orientational polarization field of solvent molecules.

Therefore, the total energy of the whole system is written as,

$$E_{\mathrm{tot}}^{\mathrm{GS}}=E_{\mathrm{qm}}^{\mathrm{GS}}[n]+E_{\mathrm{es}}[\rho ]+E_{\mathrm{sr}}[\{n_{\alpha }\},n]+E_{\mathrm{kin}}+E_{\mathrm{ext}}$$ 35

where *E*_qm_^GS^[*n*] is the ground state electronic energy of the orbital-based reactive subsystem,

$$E_{\mathrm{qm}}^{\mathrm{GS}}[n]=T_{s}[n]+E_{\mathrm{xc}}[n]+\langle U_{e}|n\rangle$$ 36

and we use Dirac’s bracket notation throughout this work

⟨x|y⟩=∫drx(r)y(r)

$$\left\langle x|\hat{O}|y\rangle =\iint drdr^{\prime }x(r)\hat{O}(r-r^{\prime })y(r^{\prime }\right)$$ 37

*E*_es_[ρ] is the classical electrostatic energy between all charged particles,

$$E_{\mathrm{es}}[\rho ]=\frac{1}{2}\langle \rho |\hat{G}|\rho \rangle$$ 38

with $\hat{G}(r-r^{\prime })=\frac{1}{4\pi \epsilon |r-r^{\prime }|}$ being the Green function of the Poisson equation, and ϵ the electronic permittivity of the dielectric media, which should be distinguished from the total permittivity that further includes the inertial permittivity associated with orientational polarization of solvent molecules.

*E*_sr_[{*n*_α_}, *n*] is the short-range, pairwise interactions between electrons, ions and solvent molecules,

$$E_{\mathrm{sr}}[\rho ]=\sum_{\alpha =c,a,s}\langle n|{\hat{V}}_{\alpha }|n_{\alpha }\rangle +\frac{1}{2}\sum_{\alpha ,\gamma =c,a,s}\langle n_{\alpha }|{\hat{W}}_{\alpha \gamma }|n_{\gamma }\rangle$$ 39

where *V̂*_α_(**r – r** ^′^) describes the short-range interactions between electrons and particles in the orbital-free environment, of which the number density is collectively denoted as *n*_α_. *Ŵ*_αγ_(**r – r** ^′^) are the short-range pairwise interactions between particles of α and γ types in the orbital-free environment. It is understood that the summation sign runs over ions (α, γ = a, c) and solvent molecules (α, γ = s).

*E*_kin_ is the classical kinetic energy of ions and solvent,

$$E_{\mathrm{kin}}=\sum_{\alpha =c,a,s}\sum_{i=1}^{N_{\alpha }}\frac{(p_{\alpha ,i}{)}^{2}}{2M_{\alpha }}$$ 40

where *M*_α_ is the mass of particles of type α.

*E*_ext_ is the energy term corresponding to external potentials applied on ions and solvent,

$$E_{\mathrm{ext}}=\sum_{\alpha =c,a,s}\langle U_{\alpha }|n_{\alpha }\rangle$$ 41

It is understood that the summation sign runs over ions and solvent molecules.

### Grand-Canonical Ensemble

After obtaining the total energy of the canonical ensemble, we proceed to treat the grand canonical ensemble. The electrochemical potentials of electrons, adsorbates, ions, and solvent molecules are denoted μ̃_e_, μ̃_A_, {μ̃_α_} and μ̃_s_. The number of metal atoms in the orbital-based reactive subsystem is fixed.

The partition function of the grand canonical ensemble is approximated as,

$$\Xi =\Xi_{e}\Xi_{n}$$ 42

where the electronic partition function over the wave function space is

$$\Xi_{e}=\sum_{N_{e}=1}^{\infty }e^{\beta {\tilde{\mu }}_{e}N_{e}}\sum_{\Psi_{N_{e}}}e^{-\beta \langle \Psi_{N_{e}}|{\hat{T}}_{e}+{\hat{V}}_{\mathrm{ee}}+{\hat{U}}_{e}|\Psi_{N_{e}}\rangle }$$ 43

where *N*_e_ = ∫ *d* **r** *n*(**r**) is the total number of electrons, Ψ_*N*_e__ is the electronic wave function of the system with *N*_e_ electrons.

A basic relationship in statistical thermodynamics relates the partition function of a grand canonical ensemble Ξ to its grand potential Ω

$$\Omega =-k_{B}T\log\;\Xi$$ 44

Since we have known the grand potential of the electronic part,

$$\Omega_{e}=T_{s}[n]+E_{\mathrm{xc}}[n]+U_{e}[n]-TS_{e}[n]-{\tilde{\mu }}_{e}\int drn(r)$$ 45

the electronic partition function is obtained as,

$$\Xi_{e}=e^{-\beta \{T_{s}[n]+E_{\mathrm{xc}}[n]+U_{e}[n]-TS_{e}[n]-{\tilde{\mu }}_{e}\int drn(r)\}}$$ 46

The nuclear partition function over the momentum and coordinate space is,

$$\Xi_{n}=\prod_{\alpha =A,s,c,a}\sum_{N_{\alpha }=1}^{\infty }\frac{e^{\beta {\tilde{\mu }}_{\alpha }N_{\alpha }}}{N_{\alpha }!}\prod_{i=1}^{N_{\alpha }}\frac{\int dR_{\alpha ,i}\int d\Omega_{\alpha ,i}}{\lambda_{\alpha }^{3}}\Theta_{\mathrm{es}}^{\left(2\right)}\Theta_{\mathrm{sr}}^{\left(2\right)}\Theta_{\mathrm{ext}}^{\left(1\right)}$$ 47

where λ_α_ is the thermal de Broglie wavelength, resulted from the integration over the momentum space of classical ions and solvent molecules, *d* **Ω**_α, *i*_ is the solid angle differential

$$d\Omega_{\alpha ,i}=\frac{\sin\theta_{\alpha ,i}}{4\pi }d\theta_{\alpha ,i}d\varphi_{\alpha ,i}$$ 48

where $\theta_{\alpha ,i}\in \left[0,\frac{\pi }{2}\right]$ is the colatitude and φ_α, *i*_ ∈ [0,2π] the longitude of the solid angle. The denominator of 4π normalizes the volume integration of *d* **Ω**_α, *i*_ to one.

Θ_es_^(2)^ is the two-body electrostatic interaction term,

$$\Theta_{\mathrm{es}}^{\left(2\right)}=e^{-\beta /2\langle \rho |\hat{G}|\rho \rangle }$$ 49

Θ_sr_^(2)^ is the two-body short-range interaction term,

$$\Theta_{\mathrm{sr}}^{\left(2\right)}=e^{-\beta /2\sum_{\alpha ,\gamma }\left\langle n_{\alpha }\right|{\hat{W}}_{\alpha \gamma }\left|n_{\gamma }\right\rangle }$$ 50

and Θ_ext_^(1)^ is the one-body external term

$$\Theta_{\mathrm{ext}}^{\left(1\right)}=e^{-\beta \sum_{\alpha }\left\langle \{U_{\alpha }+\left\langle n\right|V_{\alpha }\right\rangle \}\left|n_{\alpha }\right\rangle }$$ 51

### Particle to Field Transformation

Till here, formalism is a density-based one since all energy terms are expressed as a function of particle densities and coordinates of the particles. The overarching objective of particle-based approaches, including Kohn–Sham DFT, molecular dynamics and Monte Carlo simulations, is to either determine the coordinates of these particles under stationary conditions or sample the trajectories of these particles under dynamic conditions.^85^ Using Hubbard–Stratonovich (HS) transformation, we could transform the two-body interaction term to one-body terms interacting with auxiliary potentials, which is a well-documented approach in the literature, see the recent book chapter by Budkov and Kalikin,^86^ and also Bruch et al.^87^ This transformation leads to field-based approaches, of which the objective is to determine the stationary distributions of auxiliary potentials or track the evolution of the auxiliary potentials.^85^

Following Budkov and Kolesnikov,^88^ we have,

$$\Theta_{\mathrm{es}}^{\left(2\right)}=e^{-\beta /2\langle \rho |\hat{G}|\rho \rangle }=\frac{\int D\varphi e^{-\beta /2\langle \varphi |{\hat{G}}^{-1}|\varphi \rangle +i\beta \langle \varphi |\rho \rangle }}{\int D\varphi e^{-\beta /2\langle \varphi |{\hat{G}}^{-1}|\varphi \rangle }}$$ 52

where φ­(**r**) is an auxiliary potential corresponding to the charge density ρ­(**r**), and *Ĝ* ^–1^(**r – r** ^′^) is the inverse Green function, defined as,

$$\int dr^{\prime \prime }{\hat{G}}^{-1}(r-r^{\prime \prime })\hat{G}(r^{\prime \prime }-r^{\prime })=\delta (r-r^{\prime })$$ 53

and *Ĝ* ^–1^(**r – r** ^′^) = – ϵ∇^2^δ­(**r – r** ^′^).

Similarly, for the short-range interaction term, we have,

$$\Theta_{\mathrm{sr}}^{\left(2\right)}=e^{-\beta /2\sum_{\alpha ,\gamma }\langle n_{\alpha }|{\hat{W}}_{\alpha \gamma }|n_{\gamma }\rangle }=\frac{\int D\psi e^{-\beta /2\sum_{\alpha ,\gamma }\langle \psi_{\alpha }|{\hat{W}}_{\alpha \gamma }^{-1}|\psi_{\gamma }\rangle +i\beta \sum_{\alpha }\langle \psi_{\alpha }|n_{\alpha }\rangle }}{\int D\psi e^{-\beta /2\sum_{\alpha ,\gamma }\langle \psi_{\alpha }|{\hat{W}}_{\alpha \gamma }^{-1}|\psi_{\gamma }\rangle }}$$ 54

where ψ_α_(**r**) is the corresponding auxiliary potential, and *Ŵ*_αγ_^–1^(**r – r** ^′^) the inverse potential, defined as

$$\int dr^{\prime \prime }\sum_{\lambda }{\hat{W}}_{\alpha \lambda }^{-1}(r-r^{\prime \prime }){\hat{W}}_{\lambda \gamma }(r^{\prime \prime }-r^{\prime })=\delta_{\alpha \gamma }\delta (r-r^{\prime })$$ 55

Unlike *Ĝ* ^–1^, *Ŵ*_αλ_^–1^ does not have an explicit expression in most cases except for the Morse potential according to Weyman et al.^89^

After the Hubbard-Stratonovich transformation, the grand partition function is now written as,

$$\Xi_{n}=ce^{-i\beta \int dr\varphi e(-n+Z_{M}n_{M})}\int D\varphi e^{-\beta /2\langle \varphi |{\hat{G}}^{-1}|\varphi \rangle }\int D\psi e^{-\beta /2\sum_{\alpha ,\gamma }\langle \psi_{\alpha }|{\hat{W}}_{\alpha \gamma }^{-1}|\psi_{\gamma }\rangle }$$

$$\prod_{\alpha =A,s,c,a}\sum_{N_{\alpha }=1}^{\infty }\frac{e^{\beta {\tilde{\mu }}_{\alpha }N_{\alpha }}}{N_{\alpha }!}\prod_{i=1}^{N_{\alpha }}\frac{\int dR_{\alpha ,i}\int d\Omega_{\alpha ,i}}{\lambda_{\alpha }^{3}}\Theta_{\alpha }^{\left(1\right)}$$ 56

where the normalizing terms in eqs 52 and 54 are included in the prefactor *c*, and the one-particle terms are now included in the last term Θ_mod_^(1)^,

$$\Theta_{\alpha }^{\left(1\right)}=e^{-\beta \sum_{\alpha }\langle \{U_{\alpha }+\langle n|V_{\alpha }\rangle +i\psi_{\alpha }+i\varphi Z_{\alpha }e\}|n_{\alpha }\rangle }$$ 57

for ions (α = *a*, *c*) and adsorbates (α = *A*), and

$$\Theta_{s}^{\left(1\right)}=e^{-\beta \langle \{U_{s}+\langle n|V_{s}\rangle +i\psi_{s}+i\varphi (p_{s}\cdot \nabla )\}|n_{\alpha }\rangle }$$ 58

for solvent molecules.

For ions and adsorbates (α = *A*, *a*, *c*), the integration over the solid angle is one, and we have

$$\overset{\infty }{\underset{N_{\alpha }=1}{\sum }}\frac{e^{{\mathrm{\mu ̃}}_{\alpha }N_{\alpha }}}{N_{\alpha }!}\overset{N_{\alpha }}{\underset{i=1}{\prod }}\frac{\int dR_{\alpha ,i}\int d\Omega_{\alpha ,i}}{\lambda_{\alpha }^{3}}\Theta_{\alpha }^{\left(1\right)}=\overset{\infty }{\underset{N_{\alpha }=1}{\sum }}\frac{e^{\beta {\overset{\sim }{\mu }}_{\alpha }N_{\alpha }}}{N_{\alpha }!}\overset{N_{\alpha }}{\underset{i=1}{\prod }}\frac{\int dR_{\alpha ,i}}{\lambda_{\alpha }^{3}}e^{-\beta \sum_{\alpha }\langle \{U_{\alpha }+\langle n|V_{\alpha }\rangle +i\psi_{\alpha }+i\varphi Z_{\alpha }e\}|n_{\alpha }\rangle }$$ 59

Noting $n_{\alpha }(r)=\sum_{i=1}^{N_{\alpha }}\delta (r-R_{\alpha ,i})$ and the indistinguishability of particles of the same species, we manipulate eq 59) into,

$$\sum_{N_{\alpha }=0}^{\infty }\frac{e^{\beta {\tilde{\mu }}_{\alpha }N_{\alpha }}}{N_{\alpha }!\lambda_{\alpha }^{3N_{\alpha }}}{\left\{\int dre^{-\beta \{U_{\alpha }+\langle n|V_{\alpha }\rangle +i\psi_{\alpha }+i\varphi Z_{\alpha }e\}}\right\}}^{N_{\alpha }}=\exp\left(\lambda_{\alpha }^{-3}e^{\beta {\tilde{\mu }}_{\alpha }}\int dre^{-\beta (U_{\alpha }+\langle n|V_{\alpha }\rangle +i\psi_{\alpha }+i\varphi Z_{\alpha }e)}\right)$$ 60

This transformation is derived from the standard Maclaurin series of the exponential function $e^{x}=\sum_{N=0}^{\infty }\frac{x^{N}}{N!}$ and *N*_α_ is summed out in the grand partition function as the grand potential should not be a function of the numbers of constituents.

Similarly, following the well-known procedure of integrating out the solid phase space for dipole moment, see, for instance, Bruch et al.,^87^ the solvent term is put into,

$$\sum_{N_{s}=1}^{\infty }\frac{e^{{\tilde{\mu }}_{s}N_{s}}}{N_{s}!}\prod_{i=1}^{N_{s}}\frac{\int dR_{s,i}\int d\Omega_{s,i}}{\lambda_{s}^{3}}\Theta_{s}^{\left(1\right)}=\exp\left(\lambda_{s}^{-3}e^{\beta {\tilde{\mu }}_{s}}\int dre^{-\beta (U_{s}+\langle n|V_{s}\rangle +i\psi_{s}+\ln\frac{\sinh(i\beta p_{s}|\varphi |)}{i\beta p_{s}|\nabla \varphi |})}\right)$$ 61

Taking together, the grand partition function is written as,

$$\Xi =\int D\varphi D\psi \exp(-\beta S[n,\varphi ,\{\psi_{\alpha }\},\{{\tilde{\mu }}_{\alpha }\}])$$ 62

with the action,

$$S[\varphi ,\{\psi_{\alpha }\},\{{\tilde{\mu }}_{\alpha }\}]=S_{\mathrm{qm}}[n]+S_{\varphi }+S_{\left\{\psi_{\alpha }\right\}}+S_{\mathrm{sp}}$$ 63

where the quantum mechanical action is,

$$S_{\mathrm{qm}}[n]=T_{s}[n]+E_{\mathrm{xc}}[n]+U_{e}[n]-TS_{e}[n]-{\tilde{\mu }}_{e}\int drn(r)$$ 64

the action corresponding to the auxiliary potential φ is

$$S_{\varphi }=\frac{1}{2}\langle \varphi |{\hat{G}}^{-1}|\varphi \rangle$$ 65

which could be transferred to,

$$S_{\varphi }=\frac{1}{2}\int dr\epsilon (\nabla \varphi {)}^{2}$$ 66

using the integration by parts formula and the condition of φ = 0 at the surfaces, see Bruch et al.^87^

The action corresponding to the auxiliary potentials {Φ_α_} is

$$S_{\left\{\psi_{\alpha }\right\}}=\frac{1}{2}\sum_{\alpha ,\gamma }\langle \psi_{\alpha }|{\hat{W}}_{\alpha \gamma }^{-1}|\psi_{\gamma }\rangle$$ 67

and the single-particle action is,

Ssp=∫driφe(−n+ZMnM)−∑α=A,c,aβ−1λα−3eβμ~α∫dre−β(Uα+⟨n|Vα⟩+iψα+iφZαe)−β−1λs−3eβμ~s∫dre−β(Us+⟨n|Vs⟩+iψs+lnsinh(iβps|φ|)iβps|∇φ|) 68

### Mean-Field Approximation to the Grand Potential

The grand potential is defined as,

$$\Omega =-\beta^{-1}\ln\;\Xi$$ 69

The electrostatic potential ϕ is defined as,^87^

$$\phi =-\frac{\delta \Omega [\rho_{\mathrm{aux}}]}{\delta \rho_{\mathrm{aux}}}=i\langle \varphi \rangle$$ 70

where Ω­[ρ_aux_] is the grand potential in the presence of an auxiliary charge density ρ_aux_. We denote the statistical average of a quantity *a* is defined as,

$$\langle a\rangle =\frac{1}{\Xi }\int D\varphi D\psi ae^{-\beta S[n,\varphi ,\{\psi_{\alpha }\},\{{\mathrm{\mu ̃}}_{\alpha }\}]}$$ 71

Similarly, we define real-valued potentials corresponding to ψ_α_,

$$\Phi_{\alpha }=i\langle \psi_{\alpha }\rangle$$ 72

Next, we adopt saddle-point approximation and solve distributions of ϕ and Φ_α_, see a pedagogical introduction by Wang.^90^ According to the saddle-point approximation, the grand partition function, which is an integral of the fluctuating potentials ϕ, {Φ_α_}, is contributed dominantly by the zone near the minimum of *S*[*n*, ϕ, {Φ_α_}, {μ̃_α_}],

$$\Omega \approx S[n,\phi ,\{\Phi_{\alpha }\},\{{\tilde{\mu }}_{\alpha }\}]$$ 73

up to a constant that is independent of *n*, ϕ, {Φ_α_}, {μ̃_α_}.

The number densities are given by,

$$n_{\alpha }=-\frac{\delta \Omega }{\delta {\overset{\sim }{\mu }}_{\alpha }}=\lambda_{\alpha }^{-3}e^{\beta {\overset{\sim }{\mu }}_{\alpha }}e^{-\beta (U_{\alpha }+\langle n|V_{\alpha }\rangle +\Phi_{\alpha }+\phi Z_{\alpha }e)}$$ 74

for α = A, c, a, and,

$$n_{s}=-\frac{\delta \Omega }{\delta {\overset{\sim }{\mu }}_{s}}=\lambda_{s}^{-3}e^{\beta {\overset{\sim }{\mu }}_{s}}e^{-\beta (U_{s}+\langle n|V_{s}\rangle +\Phi_{s})}\frac{\sinh(\beta p_{s}|\nabla \phi |)}{\beta p_{s}|\nabla \phi |}$$ 75

These {μ̃_α_} could be determined from bulk conditions *n*_α_^0^ and *n*_s_^0^, which will be discussed in the Boundary Conditions section.

The task is now to find distributions of ϕ, {Φ_α_} to minimize *S*[*n*, ϕ, {Φ_α_}, {μ̃_α_}] under prescribed conditions of {μ̃_α_}. We obtain a set of variational field equations,

$$\frac{\delta S[n,\phi ,\{\Phi_{\alpha }\},\{{\tilde{\mu }}_{\alpha }\}]}{\delta \phi }=0$$ 76

$$\left\{\frac{\delta S[n,\phi ,\{\Phi_{\alpha }\},\{{\tilde{\mu }}_{\alpha }\}]}{\delta \Phi_{\alpha }}=0\right\}$$ 77

Variational analysis translates eq 76) to a modified Poisson–Boltzmann equation,^91–93^

$$-\nabla \left(\epsilon +\frac{p_{s}n_{s}}{\left|\nabla \phi \right|}L(\beta p_{s}|\nabla \phi |)\right)\nabla \phi =(-n+Z_{M}n_{M})e+\underset{\alpha =A,a,c}{\sum }Z_{\alpha }en_{\alpha }$$ 78

where *L* is the so-called Langevin function, $L\left(x\right)=\coth\left(x\right)-\frac{1}{x}$ .

Similarly, we have the following equation for the steric potentials,

$$-\left\langle W_{\alpha \alpha }^{-1}\right|\Phi_{\alpha }\rangle -\frac{1}{2}\underset{\gamma \neq \alpha }{\sum }\left\langle W_{\alpha \gamma }^{-1}\right|\Phi_{\gamma }\rangle +n_{\alpha }=0$$ 79

The EDL is often polarized to be composed of counterions of the same type. This leads to the approximation of only considering short-range interactions between particles of the same type. In this case, eq 79 is simplified to,

$$-\langle W_{\alpha \alpha }^{-1}|\Phi_{\alpha }\rangle +n_{\alpha }=0$$ 80

Following Weyman et al.,^89^ we consider only the repulsive part of the Morse potential,

$$W_{\alpha \alpha }(r)=\sigma_{\alpha \alpha }\exp(-2\xi_{\alpha \alpha }(r-d_{\alpha \alpha }))$$ 81

where σ_αα_, ξ_αα_, and *d*_αα_ are Morse parameters.

Weymann et al. gives,^89^

$$W_{\alpha \alpha }^{-1}(r)=f_{\alpha \alpha }[\nabla^{4}\delta (r)-8\xi_{\alpha \alpha }^{2}\nabla^{2}\delta (r)+16\xi_{\alpha \alpha }^{4}\delta (r)]$$ 82

with *f*_αα_ = (16πσ_αα_ξ_αα_ exp (2*d*_αα_ξ_αα_))^−1^.

The fourth-order differential equation for Φ_α_ is given by,

$$\nabla^{4}\Phi_{\alpha }-8\xi_{\alpha \alpha }^{2}\nabla^{2}\Phi_{\alpha }+16\xi_{\alpha \alpha }^{4}\Phi_{\alpha }=f_{\alpha \alpha }^{-1}n_{\alpha }$$ 83

It is noted that eq 83 has a real screening length $\lambda =\frac{1}{2\xi_{\alpha \alpha }}$ , originating from the pairwise, short-range ionic interaction. There exist other approaches to describe ionic correlation effects. For example, the Bazant-Storey-Kornyshev (BSK) theory^94^ phenomenologically describes ionic correlation using a fourth-order modified Poisson–Boltzmann equation. Similarly, built upon classical density functional theory, the interfacial layering theory of de Souza et al.^2^ expresses the electrostatic free energy in terms of a smeared charge density expansion. This approach systematically incorporates both second- and fourth-order spatial derivatives into the dielectric screening operator, allowing for more oscillator modes to be included. In short, both treatments capture ionic correlation effects by introducing higher-order dielectric response. In contrast with these two approaches, our treatment transforms ion–ion pairwise short-range interaction directly into the steric potential governed by an additional fourth-order PDE.

In short summary, the equilibrium structure of the metal-solution interfaces is now determined by three interconnected controlling equations: (1) the Kohn–Sham equations in eqs 27 and 28 for electron density, (2) the modified Poisson–Boltzmann equation in eq 78 for electrostatic potential, and (3) the high-order differential equation in eq 83 for the steric potential. The boundary conditions to close these controlling equations are specified in the next section.

### Boundary Conditions

Let us consider a metal-solution interface in a three-dimensional space with Cartesian coordinates. Let *x* be the direction perpendicular to the metal-solution interface, and *y* and *z* the other two directions in which the system is symmetrical. Therefore, the potentials ϕ and Φ_α_ and the wave functions ψ_*i*_ are symmetrical in all boundaries in *x*, *y* directions, denoted ∂Ω_*y*, *z*_,

$$\nabla \phi {|}_{\partial \Omega_{y,z}}=0$$ 84

$$\nabla \Phi_{\alpha }{|}_{\partial \Omega_{y,z}}=0$$ 85

$$\nabla \psi_{i}{|}_{\partial \Omega_{y,z}}=0$$ 86

We place the metal on the left side and denote the left boundary in the *x* dimension as ∂Ω_*x*,*L*_. The electrolyte solution is placed on the right side and the right boundary in the *x* dimension is denoted as ∂Ω_*x*,*R*_. Due to the symmetric arrangement along ∂Ω_*x*,*L*_, we have the following symmetric boundary conditions

$$\nabla \phi {|}_{\partial \Omega_{x,L}}=0$$ 87

$$\Phi_{\alpha }{|}_{\partial \Omega_{x,L}}=0$$ 88

$$\nabla^{2}\Phi_{\alpha }{|}_{\partial \Omega_{x,L}}=0$$ 89

$$\nabla \psi_{i}{|}_{\partial \Omega_{x,L}}=0$$ 90

We need two boundary conditions for Φ_α_ that is governed by a fourth-order partial differential equation. Equations 88 and 89 are natural as there are no electrolyte particles in the metal phase.

The electrolyte solution is thick enough to reach bulk conditions at ∂Ω_*x*,*R*_. We set the reference for the electric potential at ∂Ω_*x*, *R*_, namely,

$$\phi {|}_{\partial \Omega_{x,R}}=0$$ 91

We assume Φ_α_ to be uniform in the electrolyte solution bulk, namely,

$$\nabla \Phi_{\alpha }{|}_{\partial \Omega_{x,R}}=\nabla^{2}\Phi_{\alpha }{|}_{\partial \Omega_{x,R}}=0$$ 92

Therefore, the value of Φ_α_ at ∂Ω_*x*, *R*_ is determined from the controlling eq 83,

$$\Phi_{\alpha }{|}_{\partial \Omega_{x,R}}=\frac{1}{16\xi_{\alpha \alpha }^{4}f_{\alpha \alpha }}n_{\alpha }^{0}$$ 93

with *n*_α_^0^ the bulk concentration.

The probability of finding metal electrons at ∂Ω_*x*, *R*_ is zero,

$$\psi_{i}{|}_{\partial \Omega_{x,R}}=0$$ 94

Having specified the conditions of the electrolyte solution, we can determine μ̃_α_ from eqs 74 and 75,

$${\tilde{\mu }}_{\alpha }=\beta^{-1}\ln(n_{\alpha }\lambda_{\alpha }^{3})+(U_{\alpha }+\Phi_{\alpha }){|}_{\partial \Omega_{x,R}}$$ 95

for α = A, s, c, a.

### Proof-of-Concept Example of the Fourth Scheme

Here, we present a proof-of-concept implementation of the fourth scheme, where metal electrons are described using Kohn–Sham DFT and the electrolyte solution using statistical field theory. The other two dimensions of the metal-solution interface are considered to be uniform and only the perpendicular dimension x is calculated, see more details in the method section.

Figure 2 presents numerical results at Fermi energy *E*_F_ = – 2 eV. Figure 2a shows the first five electron energy levels *E*_*i*_ with increasing energy gaps. Figure 2b shows the corresponding normalized wave functions ψ̅_*i*_. The wave functions for all energy levels are constrained to zero at the right boundary in the x-dimension. For visual clarity, a vertical offset is applied to each ψ̅_*i*_ along the *y*-axis. Figure 2d displays the cumulative dimensionless electron density *n̅*_e_ (*E*_*i*_) for the first i energy levels. By summing up the contributions of all energy levels, we obtain the electron density result (black, solid line in Figure 2d). Friedel oscillations are observed arising from the quantum interference effects in wave functions.

**Figure 2:**
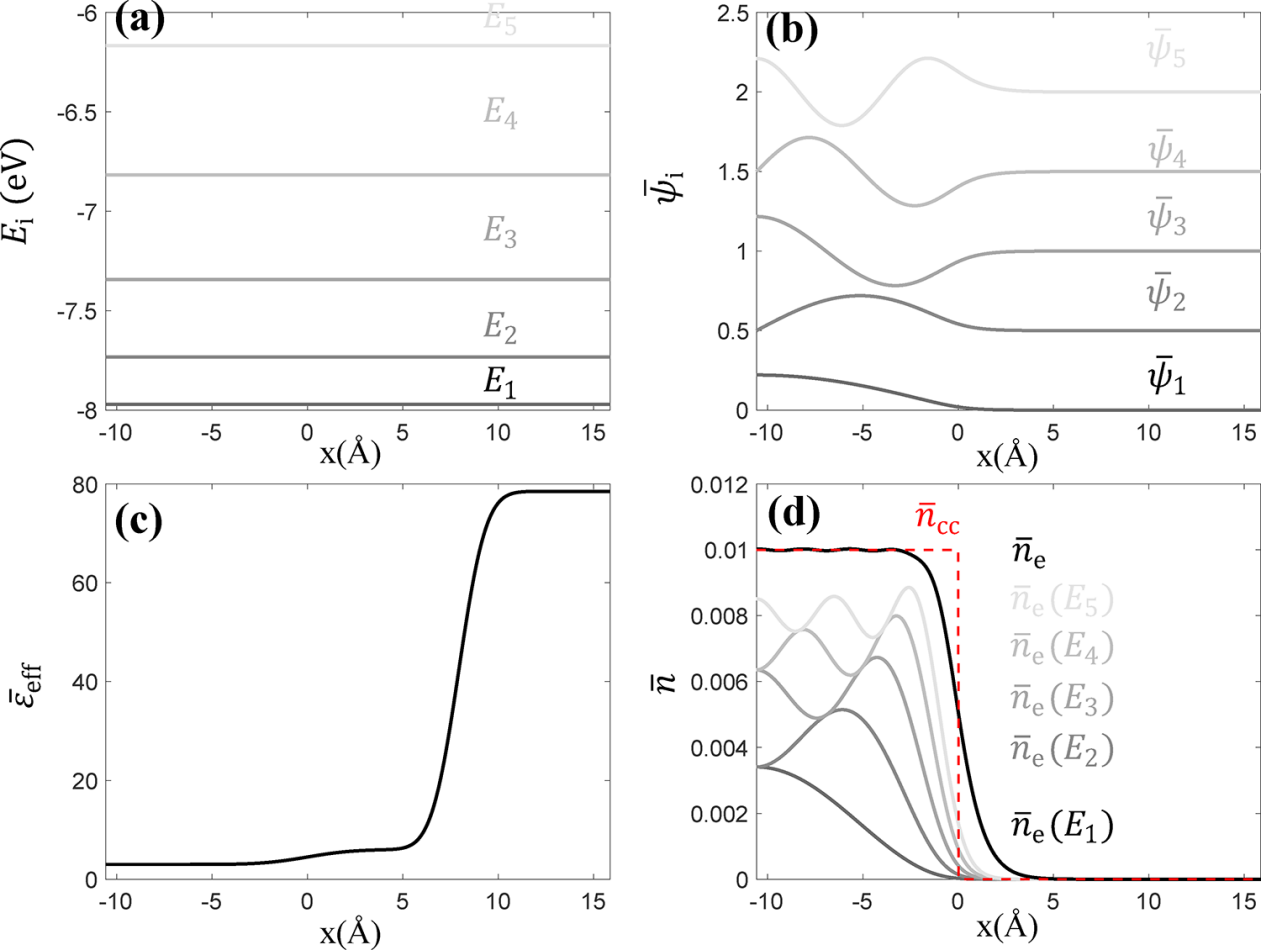
Basic numerical results of the proof-of-concept model at *E*_F_ = – 2 eV: (a) distributions of the first five electron energy levels *E*_*i*_, (b) distributions of first five normalized wave functions ψ̅_*i*_ (offset for visualization), (c) spatial distribution of relative effective dielectric permittivity ${\overset{®}{\epsilon }}_{\mathrm{eff}}=\frac{\epsilon_{\mathrm{eff}}}{\epsilon_{0}}$ , and (d) dimensionless distributions of metal nuclei density and electron density with sub-band contributions.

Figure 3 shows the numerical results for cation/anion distributions, and corresponding steric potentials at μ̃_e_ = – 2.0 eV, where the electrode is highly negatively charged with cation accumulation at the interface. This interfacial accumulation is driven by the negative surface electric potential (see Figure 3c), but is partially suppressed due to the steric repulsion, as shown in Figure 3b.

**Figure 3:**
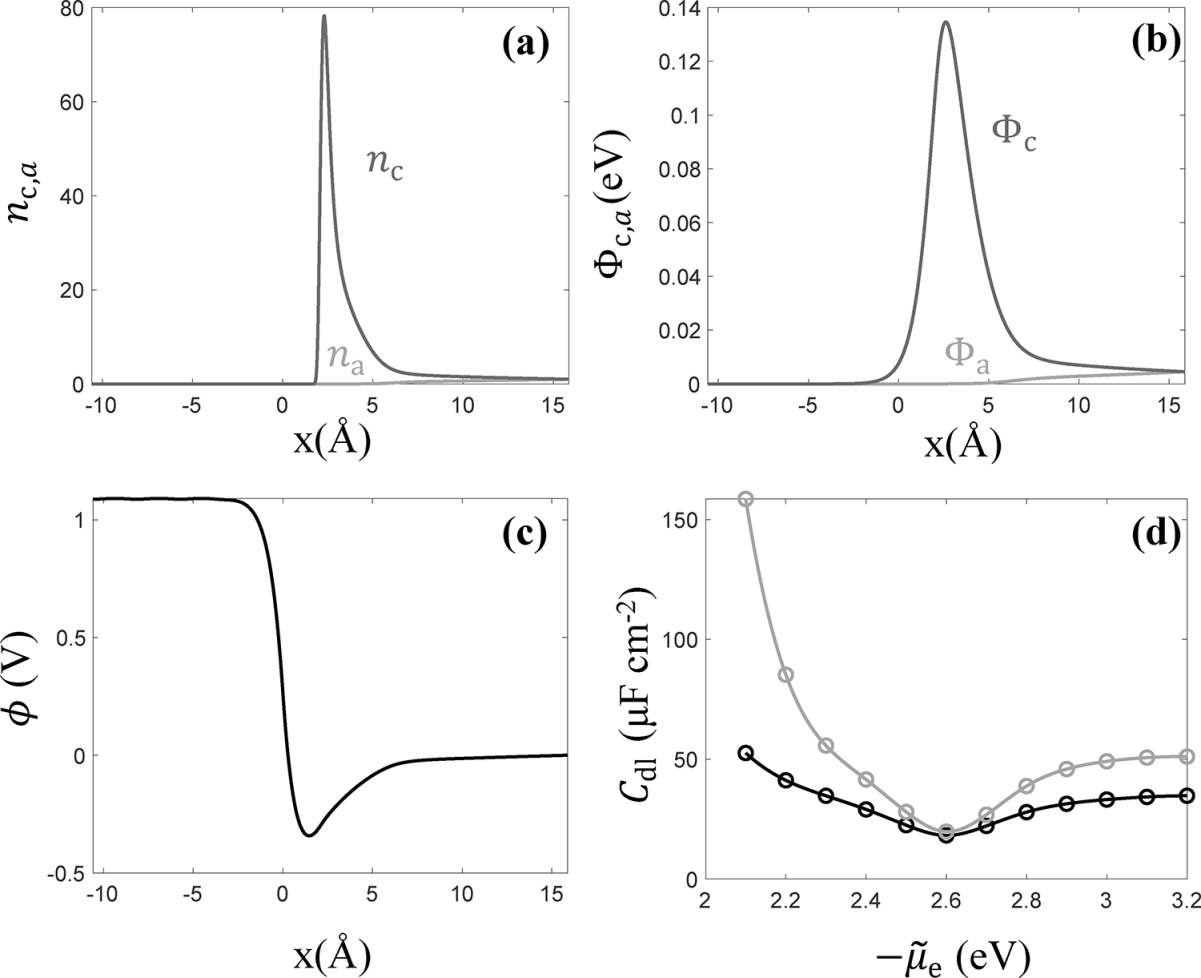
Basic results of 1D EDL model at μ̃_e_ = −2 eV: (a) distribution of cation/anion density referenced to the solution bulk density, (b) steric potential distribution of cations and anions, (c) distribution of electric potential, and (d) double layer capacitance curves (gray: without steric effect, black: with steric effect).

In this case, the surface free charge density for the EDL is defined as

$$\sigma_{\mathrm{free}}=\int_{0}^{+\infty }(n_{a}-n_{c})e_{0}dx=\frac{e_{0}}{a_{0}^{2}}\int_{0}^{+\infty }({\overset{-}{n}}_{a}-{\overset{-}{n}}_{c})d\overset{-}{x}$$

where the *x* range is from metal bulk to solution bulk and the second term is the definition in dimensionless form.

When calculating at a series of electrode potentials μ̃*_e_*, double layer capacitance *C*_dl_ curve can be obtained by differentiating surface free charge density σ_free_ with electrochemical potential of electrons, i.e. $C_{\mathrm{dl}}=-e_{0}\frac{\partial \sigma_{\mathrm{free}}}{\partial {\tilde{\mu }}_{e}}$ . The minimum of *C*_dl_ here indicates the potential of zero charge. Figure 3d shows the double layer capacitance curves in the range of μ̃*_e_* from −2.1 to −3.2 eV. It gives the potential of zero charge at μ̃_e_ = −2.6 eV. Compared with the model result without steric effect, *C*_dl_ decreases due to steric repulsion between ions of the same type. The suppression is especially pronounced when the electrode is highly charged due to strong steric potentials at interface, presenting a less sharp “V-shaped” *C*_dl_ curve, as shown in Figure 3d.

## Discussion and Concluding Remarks

Five approximation schemes can be drawn out from the preceding theoretical analysis. Figure 1 summarizes these five schemes, and Figure 1b compares them in four dimensions, namely, accuracy, system consistency, computational efficiency and transferability. By system consistency we mean how well the scheme treats the grand canonical nature of EDL and the statistical sampling of the electrolyte solution. We divide them into particle- and field-based approaches, c.f., a comparison between these two approaches by Lequieu.^85^ Particle-based approaches simulate the system directly based on particle–particle interactions. In comparison, field-based approaches introduce a fluctuating auxiliary field, transforming particle–particle interactions into particle-field interactions. The key advantage of field-based approaches to model EDL lies in preserving the system consistency and increasing the simulation efficiency at the cost of less atomic structure information.

The first scheme describes both solid electrode and electrolyte solution on the level of Kohn–Sham DFT. The approximations involved in this scheme include the Born–Oppenheimer approximation to decouple the wave functions of atom nuclei and electrons, and the approximations in the exchange-correlation functional of many interacting electrons. The controlling equation of this scheme is the Kohn–Sham equation expressed in eq 17, supplemented by relationships expressed in eqs 19–22. As shown in Figure 1b, this scheme has the highest accuracy among the five schemes. However, since it belongs to the type of particle-based simulation and since it requires solving wave functions at a cost that scales cubically with the number of atoms, it is usually employed to smaller systems with hundreds of atoms. Therefore, it is nontrivial to sufficiently sample the electrolyte solution statistically, and to simulate an open system with varying particle numbers during a simulation. We refer interested readers in simulating open systems to a comprehensive review by Delle Site and Praprotnik.^95^ This scheme is appealing for modeling interfacial chemical reactions at atomic/molecular scale. This scheme is generally applicable for interfaces formed at both metals and semiconductors in contact with various electrolyte solutions, such as water-in-salt electrolytes, polymer electrolytes, or ionic liquids.

The second scheme uses OF-DFT to describe the electrolyte solution, while the reactive metal surface remains treated using KS-DFT as in the first scheme. The set of controlling equations include eq 27 for the orbital-based reactive part, and eq 30 for the orbital-free nonreactive environment. An OF-DFT treatment of the electrolyte solution significantly reduces the computational cost of the system because the electrolyte solution has a much longer screening length and hence much thicker in the simulation cell than the solid electrode. Computational efficiency comes at the cost of lower accuracy, however, due to the approximate kinetic energy functional in the OF-DFT.^60–63^ The errors of OF-DFT also spread to the reactive metal–adsorbate subsystem via introducing an auxiliary potential in the KS-DFT due to the nonadditivity of kinetic energy functional.^76,79^ However, since no chemical reaction occurs in the electrolyte solution, we might hope that the accuracy issue of OF-DFT is not too severe thanks to error cancellation. In terms of system consistency, the second scheme remains a particle-based approach, retaining the challenge of simulating grand-canonical open systems. In terms of applications, provided with careful benchmarks, the second scheme has no apparent restrictions on the type of electrode and electrolyte materials.

The third scheme avoids calculating the electronic structure of the electrolyte solution; instead, it uses frozen densities for ions and solvent molecules to describe two-body interactions. This leads to a major reduction in the computational cost. However, a frozen density description eliminates the capability of considering dynamic polarization in the electron density of the electrolyte solution, impairing the accuracy of describing the electrolyte solution.^96^ A key difference between the second and third scheme is that the electron density in the electrolyte solution is solved using OFDFT in the second scheme while it is not solved but directly assigned in the third scheme. Hodak, Lu and Bernholc (HLB) developed a simulation method for biological systems, integrating KS-DFT for the chemically active part, including the first solvation shell, of the system and frozen-density orbital-free DFT for the rest.^78^ As an earlier example of the third scheme, the HLB method aims at resolving the disadvantage of QM/MM methods in treating the interactions between the chemically active core and the environment, improving over the oft-used electrostatic-embedding method. Like the second scheme, this scheme enjoys wide applications, provided systematic benchmarks of the OFDFT used.

Distinct from the first three schemes, which are density functional theoretic (DFT) approaches, the fourth scheme represents a hybrid density-potential functional theoretic approach (DPFT). The essential difference between DFT and DPFT lies in the status of electrostatic potential compared to density. In particle-based approaches, charge density of nuclei is predetermined at each (imaginary) time step and the electrostatic potential becomes a dependent function, governed by the Gauss’s law, of the electron density distribution. On the contrary, in a hybrid particle-field based approach, the electrostatic potential is a primal variable that has the equal status of the electron density.

Switching from a particle-based approach to a hybrid particle-field approach, we have changed the status of electrostatic potential from a dependent variable of electron density to an independent variable. This essence of this change lies in the Hubbard-Stratonovich transformation, where the fluctuating potential is introduced as an independent degree of freedom in the integration expressed in eq 52. We note that the potential of DPFT could be plural. For instance, additional auxiliary potentials corresponding to the short-range interactions between electrolyte particles are introduced in eq 54. As an additional note, DPFT is not limited to, though it can be as in the fifth scheme, a pure orbital-free method.

DPFT shares the same spirit with the hybrid particle-field theoretic approaches developed by Fredrickson et al. for polymer physics.^68,69,97^ As a key difference with hybrid particle-field theoretic approaches which are currently purely classical, DPFT is a semiclassical approach as it also describes electrons quantum mechanically.

The fourth scheme belongs to the group of continuum/implicit solvation models, such as the joint DFT method developed by Letchworth-Weaver and Arias.^35^ The key difference lies in the formulation of the grand potential. Previously, the grand potential was constructed with a higher degree of empiricism, namely, by adding a quantum mechanical part and a classical part for the electrolyte solution that is usually described on the level of (modified) Poisson–Boltzmann theory. In addition, a solvation cavity is introduced to account for short-range repulsion between the quantum mechanical part and the electrolyte solution, see discussions in Schwartz et al.^28^ and Ringe et al.^29^ In contrast, a rigorous statistical treatment of the degrees of freedom of nuclei, including both long-range electrostatic interactions and short-range interactions, is given in the fourth scheme of the present work. Auxiliary potentials Φ_α_, governed by fourth-order partial differential equations, are introduced to describe the short-range interactions. Consequently, crowding effect of ions can be captured in the present scheme. Therefore, it is appealing for simulating EDLs in highly concentrated solutions such as ionic liquids and water-in-salt electrolyte. Similarly, since the polymer electrolyte can also be described in terms of both long-range Colomb interaction and short-range interactions between chains,^98^ the fourth scheme is thus generally applicable for polymer electrolytes.

Though the DOF of the electrolyte solution has been averaged out in the fourth scheme, expensive KS-DFT limits the fourth scheme to small systems with less than hundreds of atoms in the metal–adsorbate part. Its applications include metal slabs and clusters, but nanoparticle catalysts are beyond reach. One can further reduce the computation cost by employing OF-DFT to describe most metal atoms that are not directly involved in reactions. This hybrid KS/OF-DFT approach could, in principle, allow us to simulate the reactive EDL at nanoparticles with a decent accuracy-efficiency balance.

## Methods

An in-house numerical scheme of consistently solving Kohn–Sham DFT and modified Poisson–Boltzmann equation will be published elsewhere.^99^ The slowly varying limit of Perdew–Burke–Ernzerhof (PBE) exchange-correlation potential is used in Kohn–Sham DFT, and steric potentials for cation–cation pairs Φ_c_ and anion–anion pairs Φ_a_ are considered.

The proof-of-concept model is implemented in MATLAB R2024b. Finite Difference Method is used to calculate the second-order PDE in Kohn–Sham equation. To solve the modified PB equation self-consistently, we decompose the fourth-order partial differential equation (PDE) for steric potentials in eq 83 into two second-order PDEs:

$$\nabla {®}^{2}{\overset{-}{\Phi }}_{\alpha }={\overset{-}{\zeta }}_{\alpha }$$

$${\overset{®}{\nabla }}^{2}{\overset{®}{\zeta }}_{\alpha }-8{\overset{®}{\xi }}_{\alpha \alpha }^{2}{\overset{®}{\zeta }}_{\alpha }+16{\overset{®}{\xi }}_{\alpha \alpha }^{4}{\overset{®}{\Phi }}_{\alpha }={\overset{®}{f}}_{\alpha \alpha }^{-1}{\overset{®}{n}}_{\alpha }$$

where α = a, c for anions and cations, respectively.

Five variables Φ̅_a_, ζ̅_a_, Φ̅_c_, ζ̅_c_, ϕ̅ in the modified PB equation (eq 78) are introduced. Using the Newton–Raphson iteration method,^99^ the variables can be solved simultaneously by constructing Jacobian matrix of size 5*N* × 5*N*. From a numerical perspective, though the order decomposition increases the matrix size, it makes the matrix more diagonally dominant and sparse, saving the computational cost. At each step, the variable matrix is updated with a weight factor *w* = 0.6 to avoid overshooting and enhance convergence stability.

The toy model is designed to demonstrate the feasibility of the fourth scheme. The parameters are not obtained from more accurate methods or experimental sources but are assigned with reasonable values. However, for a specific solid–liquid interface, the parameters can be obtained from the following two ways: (1) benchmarking with experimental data such as the differential double-layer capacitance curves, (2) benchmarking with high-level computations such as ab initio molecular dynamics simulation. Examples of benchmarking can be found in our previous studies^54,55,100^

*L*_M_ and *L*_S_ denote dimensionless thicknesses of the metal (from bulk to edge) and electrolyte solution, respectively. Here, we use *L*_M_ = 20 and *L*_S_ = 30, normalized to the Bohr radius *a*_0_ = 0.529 Å. For the metal, the dimensionless metal nuclei *n̅*_M_ = *n*_M_ · *a*_0_^3^ is set to 0.01 with valence of 1, namely, *Z*_M_ = 1. For the electrolyte solution, we use *n*_c, a_^0^ = 100 mM, *Z*_c_ = 1, *Z*_a_ = −1, representing 1–1 electrolyte, such as KF, with a 100 mM bulk concentration. Solvent and adsorbates are not considered explicitly in this prototypical example. We thus replace the term $\epsilon +\frac{p_{s}n_{s}}{\left|\nabla \varphi \right|}L(\beta p_{s}|\nabla \varphi |)$ in eq 78 with a field-independent ϵ_eff_. The spatial distribution for ϵ_eff_ with respect to vacuum permittivity ϵ_0_ is shown in Figure 2c. At metal bulk, ϵ_eff_ = 3ϵ_0_. It slightly increases in the metal-solution interfacial region and reaches 78.5 ϵ_0_ to simulate aqueous solvent.

Short-range interactions between electrons and ions ⟨*n* | *V*_α=c, a_⟩ and between ions of the same type *W*_αα=cc_ for cations and *W*_αα=aa_ for anions are considered. Similar to the expression in eq 81, ⟨*n* | *V*_α=c, a_⟩ is modeled using the repulsive term of Morse potential, i.e., ⟨*n* | *V*_α=c, a_⟩ = σ_c, a_ exp (− 2ξ_c, a_(*r* – *d*_c, a_)). Here we set σ_c, a_ = 0.5/6 eV, β_*c*, *a*_ = 1/*a*_0_, *d*_c, a_ = 4*a*_0_. As for *W*_αα=cc, aa_, we use σ_cc, aa_ = 0.05 eV, β_cc, aa_ = 0.5/*a*_0_, *d*_cc, aa_ = 6*a*_0_. The equilibrium distance between ion pairs *d*_cc, aa_ is set to be larger than the equilibrium gap between ions and metal electrode *d*_c, a_.

This proof-of concept example simulates the EDL under fixed Fermi level, equivalently under constant electrode potentials. The voltage applied onto the electrode is a component of the electrochemical potential of electrons, namely, the Fermi level. In other words, the external potential is embedded into the Fermi level and thus implicitly considered.
